# Mice lacking collapsin response mediator protein 1 manifest hyperactivity, impaired learning and memory, and impaired prepulse inhibition

**DOI:** 10.3389/fnbeh.2013.00216

**Published:** 2013-12-27

**Authors:** Naoya Yamashita, Aoi Takahashi, Keizo Takao, Toshifumi Yamamoto, Pappachan Kolattukudy, Tsuyoshi Miyakawa, Yoshio Goshima

**Affiliations:** ^1^Department of Molecular Pharmacology and Neurobiology, Yokohama City University Graduate School of MedicineYokohama, Japan; ^2^Section of Behavior Patterns, Center for Genetic Analysis of Behavior, National Institute for Physiological SciencesOkazaki, Japan; ^3^Genetic Engineering and Functional Genomics Group, Frontier Technology Center, Graduate School of Medicine, Kyoto UniversityKyoto, Japan; ^4^Core Research for Evolutional Science and Technology (CREST), Japan Science and Technology AgencyKawaguchi, Japan; ^5^Laboratory of Molecular Psychopharmacology, Graduate School of Nanobiosciences, Yokohama City UniversityYokohama, Japan; ^6^Burnett School of Biomedical Sciences, College of Medicine, University of Central FloridaOrlando, FL, USA; ^7^Division of Systems Medical Science, Institute for Comprehensive Medical Science, Fujita Health UniversityToyoake, Japan

**Keywords:** CRMP1, comprehensive behavioral test, knockout mouse, schizophrenia, mesocortical dopaminergic transmission, hyperactivity, prepulse inhibition

## Abstract

Collapsin response mediator protein 1 (CRMP1) is one of the CRMP family members that are involved in various aspects of neuronal development such as axonal guidance and neuronal migration. Here we provide evidence that *crmp1*^−/−^ mice exhibited behavioral abnormalities related to schizophrenia. The *crmp1*^−/−^ mice exhibited hyperactivity and/or impaired emotional behavioral phenotype. These mice also exhibited impaired context-dependent memory and long-term memory retention. Furthermore, *crmp1*^−/−^ mice exhibited decreased prepulse inhibition, and this phenotype was rescued by administration of chlorpromazine, a typical antipsychotic drug. In addition, *in vivo* microdialysis revealed that the methamphetamine-induced release of dopamine in prefrontal cortex was exaggerated in *crmp1*^−/−^ mice, suggesting that enhanced mesocortical dopaminergic transmission contributes to their hyperactivity phenotype. These observations suggest that impairment of CRMP1 function may be involved in the pathogenesis of schizophrenia. We propose that *crmp1*^−/−^ mouse may model endophenotypes present in this neuropsychiatric disorder.

## Introduction

Collapsin response mediator protein (CRMP) was originally identified as a signaling molecule of Semaphorin3A (Sema3A), a repulsive axonal guidance molecule (Goshima et al., 1995). CRMPs are now known to consist of five homologous cytosolic proteins, CRMP1–5. All family members are highly expressed in the developing and adult nervous system (Minturn et al., 1995; Byk et al., 1996; Hamajima et al., 1996; Wang and Strittmatter, 1996; Fukada et al., 2000; Inatome et al., 2000; Yuasa-Kawada et al., 2003). In cultured neurons, CRMPs have been shown to be involved in axon specification, elongation, and navigation, suggesting that they play multifunctional roles in neuronal development. CRMPs are present as phosphoproteins in the developing nervous system (Byk et al., 1996). CRMPs bind to tubulin heterodimer, and upon phosphorylation of CRMPs by Rho/ROCK kinase, cyclin-dependent kinase-5 (Cdk5), and glycogen synthase kinase-3β, the binding affinity of CRMPs to tubulin is lowered (Fukata et al., 2002; Uchida et al., 2005; Yoshimura et al., 2005). Thus, phosphorylation of CRMPs by these kinases is thought to play important roles in neuronal development (Yamashita and Goshima, 2012).

Several knockout mice studies have begun to reveal the *in vivo* roles of CRMPs in neuronal development and function (Charrier et al., 2006; Yamashita et al., 2006, 2007, 2011, 2012; Su et al., 2007; Quach et al., 2008; Niisato et al., 2012a,b). CRMP1 regulates neuronal cell migration, dendritic spine development, and synaptic plasticity *in vivo* (Charrier et al., 2006; Yamashita et al., 2006, 2007; Su et al., 2007). To regulate neuronal cell migration, CRMP1 is phosphorylated by Fyn, a Src-type tyrosine kinase, downstream of Reelin (Yamashita et al., 2006). Furthermore, phosphorylation of CRMP1 by Cdk5 is essential for Sema3A-induced spine development (Yamashita et al., 2007). These findings indicate that CRMP1 mediates signals from several extracellular molecules that play essential roles in neuronal network formation and synapse formation. However, the roles of CRMP1 in the regulation of higher brain functions are largely unknown.

To address this issue, we carried out a comprehensive behavioral analysis of *crmp1*^−/−^ mice. We found that they exhibited hyperactivity, impaired learning and memory, and impaired prepulse inhibition. Because these phenotypes appear to be analogous to some of the symptoms of schizophrenia, we propose that *crmp1*^−/−^ mouse is useful in exploring basic mechanisms that may contribute to some of the symptoms of schizophrenia.

## Materials and methods

### Mutant mice and experimental design

*Wild-type* (*wt*) and *crmp1*^−/−^ mice (Charrier et al., 2006) were generated by mating *crmp1*^+/−^ heterozygotes that had been backcrossed to the C57BL/6N background for at least seven generations. Genotypes of the offspring of all mutant mice were assessed with the use of polymerase chain reaction, as described previously (Charrier et al., 2006). Comprehensive behavioral test battery (Takao and Miyakawa, 2006) (except drug treatments for the prepulse inhibition test, see below) was carried out with single male mice that were at least 21 weeks of age. Raw data of the behavioral test battery, the date on which each experiment was done, and the age of the mice at the time of the experiment are shown in the mouse phenotype database (Table 1; http://www.mouse-phenotype.org/). Female mice were excluded because their behavior is influenced by the menstrual cycle. To minimize the effects of previous tests on subsequent tests, we performed the behavioral test battery in a specific order, in which the less stressful tests preceded the more stressful tests. Each behavioral test was separated from each other at least by one day. The order of the battery test and the summary of each test result were described in Table 1.

**Table 1:** **Comprehensive behavioral test battery of *crmp1*^−/−^ mice**.

**Test**	**Measurements**	**Age (w)**	**Phenotype**
1. General health and neurological screening	Body weight	21–25	↓
	Body temperature		→
	Wire hanging time		→
	Grip strengthen		→
2. Light/dark transition	Light/dark transition	21–25	→
3. Open field (Figures 1B–E)	Total distance	21–26	↑
	Vertical activity		→
	Center time		↑
	Stereotypic counts		→
4. Elevated plus maze (Figures 2A,B)	Time on center	21–26	↑
	Time on close arm		↓
5. Hot plate	Pain sensitivity	21–26	↑
6. Social interaction (novel environment)	Social behavior	22–26	→
7. Rotarod	Motor coordination	22–26	→
8. Social interaction (Crawley ver.)	Social behavior	23–27	→
9. Prepulse inhibition (Figures 4A,B)	Prepulse inhibition	23–27	↓
10. Porsolt forced swim (Figures 2C,D)	Immobility time	24–28	↓
11. Gait analysis	Locomotion pattern	24–29	→
12. Barnes maze (Figure 3B)	Special memory	25–29	→
	Memory retention		↓
13. T-maze	Working memory	32–36	→
14. Fear conditioning (Figure 3A)	Contextual learning	37–41	↓
	Cued learning		→
15. Tail suspension	Behavioral despair	39–43	→
16. Social interaction (home cage)	Social behavior	62–67	→
17. Home cage monitoring (Figure 1A)	Locomotor activity	69–75	↑

Age (w); age of weeks of subject at the beginning of each test.

→, no significant difference; ↑, increased; ↓, decreased.

Mice were housed in the standard mouse facility and fed an autoclaved diet and water. All procedures were performed according to the guidelines outlined in the institutional Animal Care and Use Committee of the Yokohama City University School of Medicine or Kyoto University Graduate School of Medicine. Throughout the experimental procedures, all efforts were made to minimize the number of animals used and their suffering.

### Twenty four-hour home cage monitoring

A system that automatically analyses behavior in home cages was used to monitor locomotor activity (O'Hara and Co., Tokyo, Japan) (Yamasaki et al., 2008). The system comprised the home cage (29 × 18 × 12 cm) and a filtered cage top, separated by a 13-cm high metal stand containing an infrared video camera attached at the top of the stand. Each *wt* or *crmp1*^−/−^ mouse was placed in an individual home cage, and behavior was monitored for 1 week. Images from each cage were captured at a rate of one frame per second. Their locomotor activity was measured by quantifying the number of pixels changed between successive frames. Analysis was performed automatically using Image HA software (O'Hara and Co.).

### Open field test

Each mouse was placed in the center of the open field apparatus (40 × 40 × 30 cm; Accuscan Instruments, Columbus, OH, USA). Total distance traveled (cm), vertical activity (rearing measured by counting the number of photobeam interruptions), time spent in the center (20 × 20 cm), the beam-break counts for stereotyped behaviors, and number of fecal boli were recorded. Data were collected for 120 min.

### Elevated plus maze

The elevated plus-maze (O'Hara and Co.) consisted of two open arms (25 × 5 cm) and two enclosed arms of the same size, with 15-cm-high transparent walls. The arms and central square were made of white plastic plates and were elevated to a height of 55 cm above the floor. To minimize the likelihood of animals falling from the apparatus, 3-mm-high plastic ledges were provided for the open arms. Arms of the same type were arranged at opposite sides to each other. Each mouse was placed in the central square of the maze (5 × 5 cm), facing one of the closed arms. Mouse behavior was recorded for 10 min. The number of entries into open and enclosed arms, and the time spent in the central square and open and enclosed arms were recorded. For data analysis, we used the following measures: the percentage of entries into the open arms, the time spent in the central square and open and enclosed arms (sec), and total distance traveled (cm). Data acquisition and analysis were performed automatically using Image EP software (O'Hara and Co.).

### Porsolt forced swim

The apparatus consisted of four plastic cylinders (20 cm in height, 10 cm in diameter). The cylinders were filled with water at 23°C up to a height of 7.5 cm. Mice were placed into the cylinders, and their behavior was recorded for 10 min. Data acquisition and analysis were performed automatically with Image PS software (O'Hara and Co.).

### Contextual and cued fear conditioning

Each mouse was placed in a test chamber (26 × 34 × 29 cm) and allowed to explore freely for 2 min. A 55-dB white noise, which served as the conditioned stimulus (CS), was presented for 30 s, followed by a mild (2 s, 0.35 mA) foot shock, which served as the unconditioned stimulus (US). Two more CS-US pairings were presented with an interstimulus interval of 2 min. Context testing was conducted in the same chamber 1 day after conditioning. Cued testing with altered context was performed in a triangular box (35 × 35 × 40 cm) made of white opaque Plexiglas and located in a different room 1 day after conditioning.

Data acquisition, control of stimuli (tones and shocks), and data analysis were performed automatically with Image FZ software (O'Hara and Co.). Images were captured at the rate of 1 frame per second. For each pair of successive frames, the area (number of pixels) corresponding to the movement of the mouse was measured. If this area was below a certain threshold (20 pixels), the behavior was judged as freezing. If the area equaled or exceeded the threshold, the behavior was considered nonfreezing. The optimal threshold (number of pixels) for judgment of freezing was based on evaluation of the behavior by human observation. Freezing that persisted for less than the defined time threshold (2 s) was not included in the analysis.

### Barnes maze

The Barnes task was performed on dry land, a white circular surface, 1.0 m in diameter, with 12 holes equally spaced around the perimeter (O'Hara and Co.). The circular open field was elevated 75 cm from the floor. A black Plexiglas escape box (17 × 13 × 7 cm), the bottom of which contained paper cage bedding, was located under one of the holes. The hole above the escape box represented the target, analogous to the hidden platform in the Morris task. The location of the target was consistent for a given mouse but randomized across mice. The maze was rotated daily, with the spatial location of the target unchanged with respect to the distal visual room cues, to prevent bias based on olfactory cues or the proximal cues within the maze. Three trials per day were conducted for six successive days. On day 7, a probe trial was performed without the escape box, to confirm that the spatial task was acquired on the basis of navigation via the distal environment room cues. A probe trial was also performed after 12 days of retention (day 19). The latency before the mouse reached the target hole, distance traveled to reach the target hole, number of errors, and time spent around each hole were recorded by video-tracking software (Image BM, O'Hara and Co.).

### Prepulse inhibition

A startle reflex measurement system (O'Hara and Co.) was used to measure startle response and prepulse inhibition. The test session began by placing a mouse in a plastic cylinder and leaving it undisturbed for 10 min. White noise (40 ms) was used as the startle stimulus for all trial types. The startle response was recorded for 140 ms (with measurement of the response every 1 ms) beginning with the onset of the prepulse stimulus. The background noise level in each chamber was 70 dB. The peak startle amplitude recorded during the 140-ms sampling window was used as the dependent variable. A test session consisted of six trial types (two types of startle stimulus only trial, and four types of prepulse inhibition trial). The intensity of the startle stimulus was 110 or 120 dB. The prepulse sound (74 or 78 dB) was presented 100 ms before the startle stimulus. Four combinations of prepulse and startle stimuli were used (74 and 110, 78 and 110, 74, and 120, and 78, and 120 dB). Six blocks of the six trial types were presented in pseudorandom order such that each trial type was presented once within a block. The average intertrial interval was 15 s (range, 10–20 s). For drug treatments, 12-week-old *crmp1*^−/−^ mice were placed in a startle reflex measurement system 5 min after intraperitoneal injection of chlorpromazine (1 mg/kg, Mitsubishi Tanabe Pharma, Osaka, Japan) or saline.

### *In vivo* microdialysis

*In vivo* microdialysis measurements of extracellular dopamine were performed in freely moving mice (Jitsuki et al., 2011). Mice were anesthetized with isoflurane and a guide cannula (AG-4; EICOM, Kyoto, Japan) was implanted stereotaxically into the medial prefrontal cortex. The coordinates were 2 mm anterior from the bregma, 0.3 mm lateral to the midline, and 1.5 mm below the surface of the brain according to the atlas of Franklin and Paxinos (Franklin and Paxinos, 2007). Two days after the surgery, a dialysis probe (AI-4-1, 1 mm membrane length; EICOM) was inserted through the guide cannula and was perfused at a flow rate of 1.0 μl/min with artificial cerebrospinal fluid (147 mM NaCl, 4 mM KCl, 1.2 mM CaCl_2_, 0.9 mM MgCl_2_). Sample collection was started after a 2-hr equilibration period. The outflow fractions were collected every 20 min. After collection of six baseline fractions, mice were treated with methamphetamine (1 mg/kg, i.p., Dainippon Sumitomo Pharma, Osaka, Japan) and sampling was continued for an additional 180 min. The amount of dopamine in the dialysis fractions was measured by high-performance liquid chromatography on a VA5-ODS column (EICOM) that was maintained at 25°C and equipped with an electrochemical detection system (ECD-300, EICOM). The changes in electric current (nA) were recorded using an integrated data processor (Chromatocorder 12; System Instruments Co., Tokyo, Japan). The dopamine concentration in the dialysate was calculated by reference to the peak area of the standard solution. The probe locations were verified histologically.

### Image analysis

The applications used for the behavioral studies (Image HA, Image EP, Image PS, Image FZ, and Image BM) were based on the NIH Image program (developed at the U.S. National Institutes of Health and available at http://rsb.info.nih.gov/nih-image) and ImageJ program (http://rsb.info.nih.gov/ij), which were modified for each test by T. Miyakawa and are available through O'Hara and Co. Image EP (Komada et al., 2008) and Image FZ are freely available at the following URL: http://www.mouse-phenotype.org/software.html

### Statistical analysis

Statistical analysis was conducted using StatView (SAS Institute, Cary, NC, USA). Data were analyzed by paired *t*-test, one-way ANOVA, or two-way repeated measures ANOVA, unless noted otherwise. Values in graphs are expressed as mean ± s.e.m. *Post-hoc* comparisons were performed using Fisher's protected least significant difference (Fisher's PLSD) multiple comparisons. Genotype or drug × time was calculated by a repeated measures ANOVA. The statistical treatment of data is described in the figure legend.

## Results

### Hyperactivity and impaired emotional behavioral phenotype of *crmp1*^−/−^ mice

To investigate the possible behavioral effects of CRMP1 deficiency, we subjected *crmp1*^−/−^ mice and their *wt* littermates to a comprehensive behavioral test battery (Takao and Miyakawa, 2006). The *crmp1*^−/−^ mice appeared healthy and showed no obvious differences in physical characteristics, as described previously (Charrier et al., 2006). A small but significant decrease in body weight of *crmp1*^−/−^ mice was observed compared to *wt* control animals [28.675 ± 1.174 g in *wt* (*n* = 8), 24.825 ± 0.709 g in *crmp1*^−/−^ (*n* = 8), *F*_(1, 14)_ = 7.875, *p* = 0.014 by one-way ANOVA]. No significant differences in neuromuscular strength (grip strength and wire hang tests) or sensorimotor reflexes (eye blink, ear twitch, whisker touch, and righting reflex tests) were apparent between *wt* and *crmp1*^−/−^ mice (Table 1).

**Figure 1:**
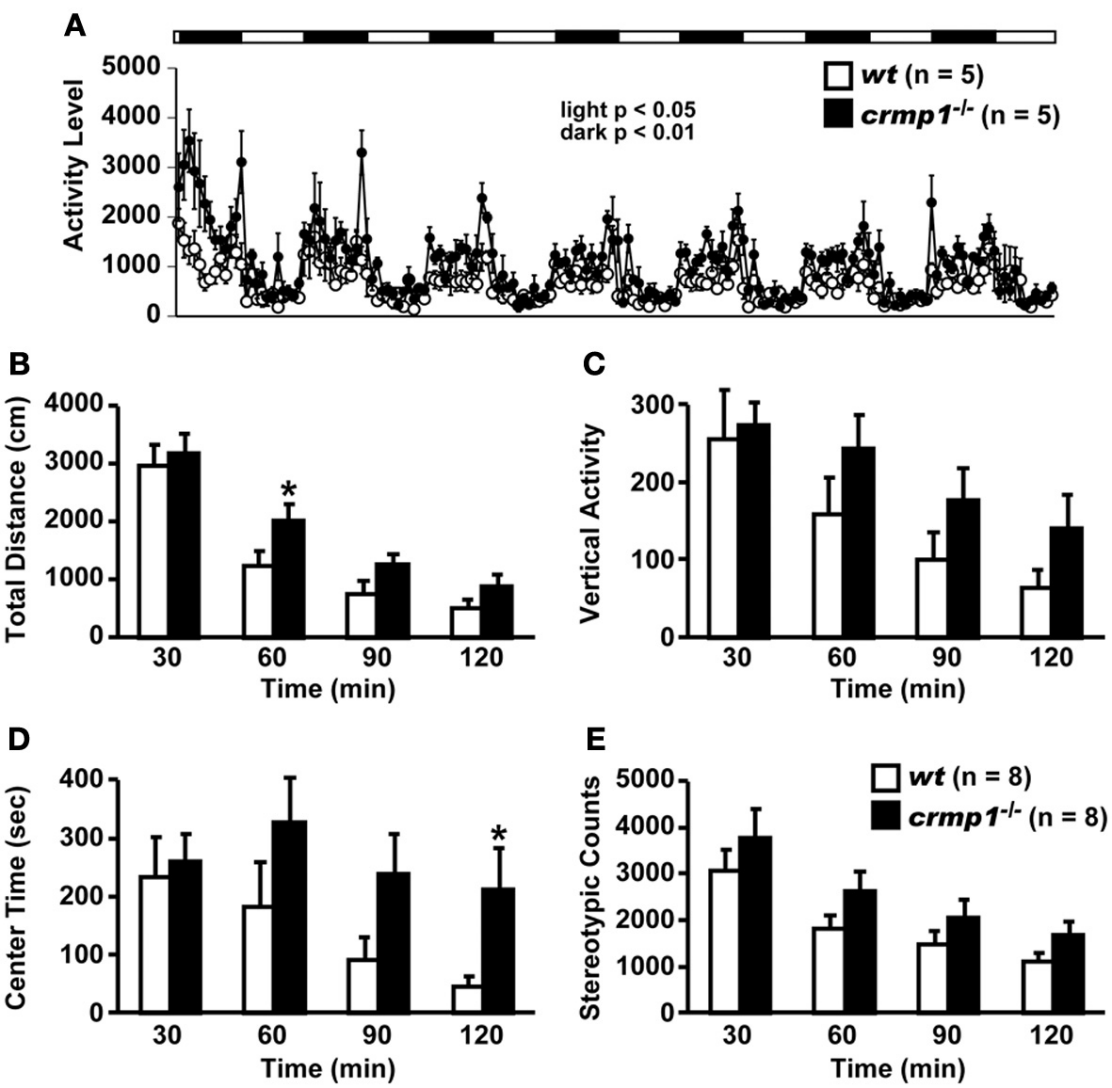
**Increased locomotor activity of *crmp1*^−/−^ mice in the 24-h home cage monitoring and the open field test**. **(A)** Locomotor activity of *wt* and *crmp1*^−/−^ mice was monitored in their home cages continuously for 1 week. The white and black areas above the graph indicate light and dark periods, respectively. **(B–E)** The open field test with total distance **(B)**, vertical activity **(C)**, center time **(D)**, and stereotypic counts **(E)**. Statistic analysis was performed every 30 min. The *p* values indicate genotype effect in two-way repeated measures ANOVA. Data are shown as mean ± s.e.m. for the indicated numbers of mice. ^*^p < 0.05.

Examination of the locomotor activity of *crmp1*^−/−^ mice in several behavioral tasks revealed a hyperactivity phenotype of the mutant animals. In 24-h home cage monitoring, the activity levels of *crmp1*^−/−^ mice were significantly higher than those of *wt* mice in both light and dark phases [Figure 1A; dark period, *F*_(1, 8)_ = 12.155, *p* = 0.0082; light period, *F*_(1, 8)_ = 7.349, *p* = 0.0266]. This hyperactivity phenotype was also observed in an open field test. In *crmp1*^−/−^ mice, the significant increases in distance traveled and time spent in the central part were observed at 30 to 60 min [*F*_(1, 14)_ = 4.941, *p* = 0.0432] and at 90 to 120 min [*F*_(1, 14)_ = 5.108, *p* = 0.0403] of each test period, when compared to *wt* mice (Figures 1B–E).

**Figure 2:**
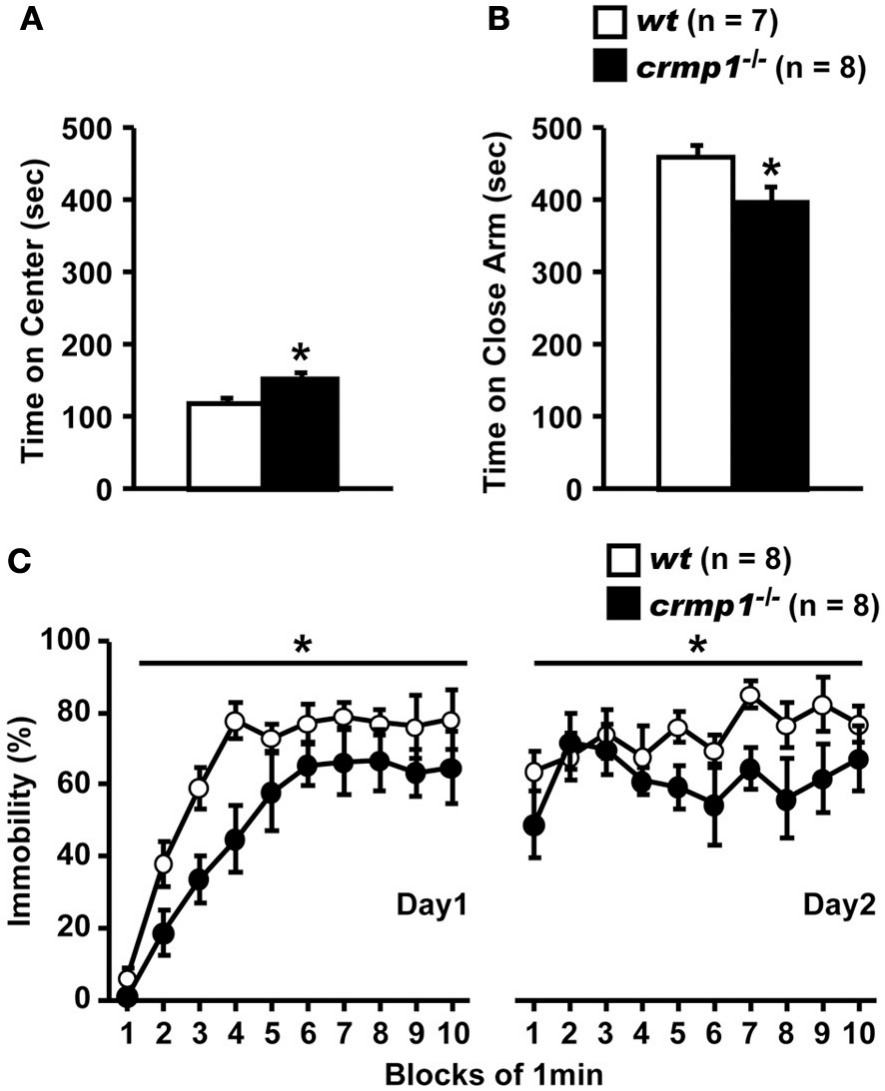
**Hyperactivity and/or impaired emotional behaviors of *crmp1*^−/−^ mice in stressful environments**. **(A,B)** The time spent on central part **(A)** and the close arms **(B)** in the elevated plus maze test. The *p* values indicate genotype effect in one-way ANOVA. **(C)** Immobility rate in the Porsolt forced swim test. The immobility time was recorded over a 10-min test period on day 1 and day 2. The *p* values indicate genotype effect in two-way repeated measures ANOVA. Data are shown as mean ± s.e.m. for the indicated numbers of mice. ^*^p < 0.05.

The hyperactivity phenotype of *crmp1*^−/−^ mice was also apparent in the elevated pulse maze test. Both *wt* and *crmp1*^−/−^ mice showed the typical pattern of favoring the closed arms. However, the *crmp1*^−/−^ mice spent more time in the central part [Figure 2A; *F*_(1, 13)_ = 6.250, *p* = 0.0266] and less time in the enclosed arms [Figure 2B; *F*_(1, 13)_ = 5.583, *p* = 0.0344] than *wt* mice did. In addition, *crmp1*^−/−^ mice showed a significant decrease in immobility time compared with *wt* mice in the Porsolt forced swim test [Figure 2C; day1, *F*_(1, 14)_ = 6.776, *p* = 0.0209; day2, *F*_(1, 14)_ = 5.459, *p* = 0.0348]. The abnormal phenotypes of *crmp1*^−/−^ mice in these two tests may reflect anxiety-related and depression-related behavior, respectively (Takao and Miyakawa, 2006). It is therefore possible that CRMP1 deficiency affects emotional behavior. Consistent with this, in the open field test, significant increases were observed in distance traveled as well as time spent in the central part, which reflects anxiety-related behavior (Figures 1B,D). These data suggest that *crmp1*^−/−^ mice exhibited hyperactivity phenotype and/or impaired emotional behavioral phenotype, which consequently affect locomotor activity of these mice.

### Impaired learning and memory of *crmp1*^−/−^ mice

**Figure 3:**
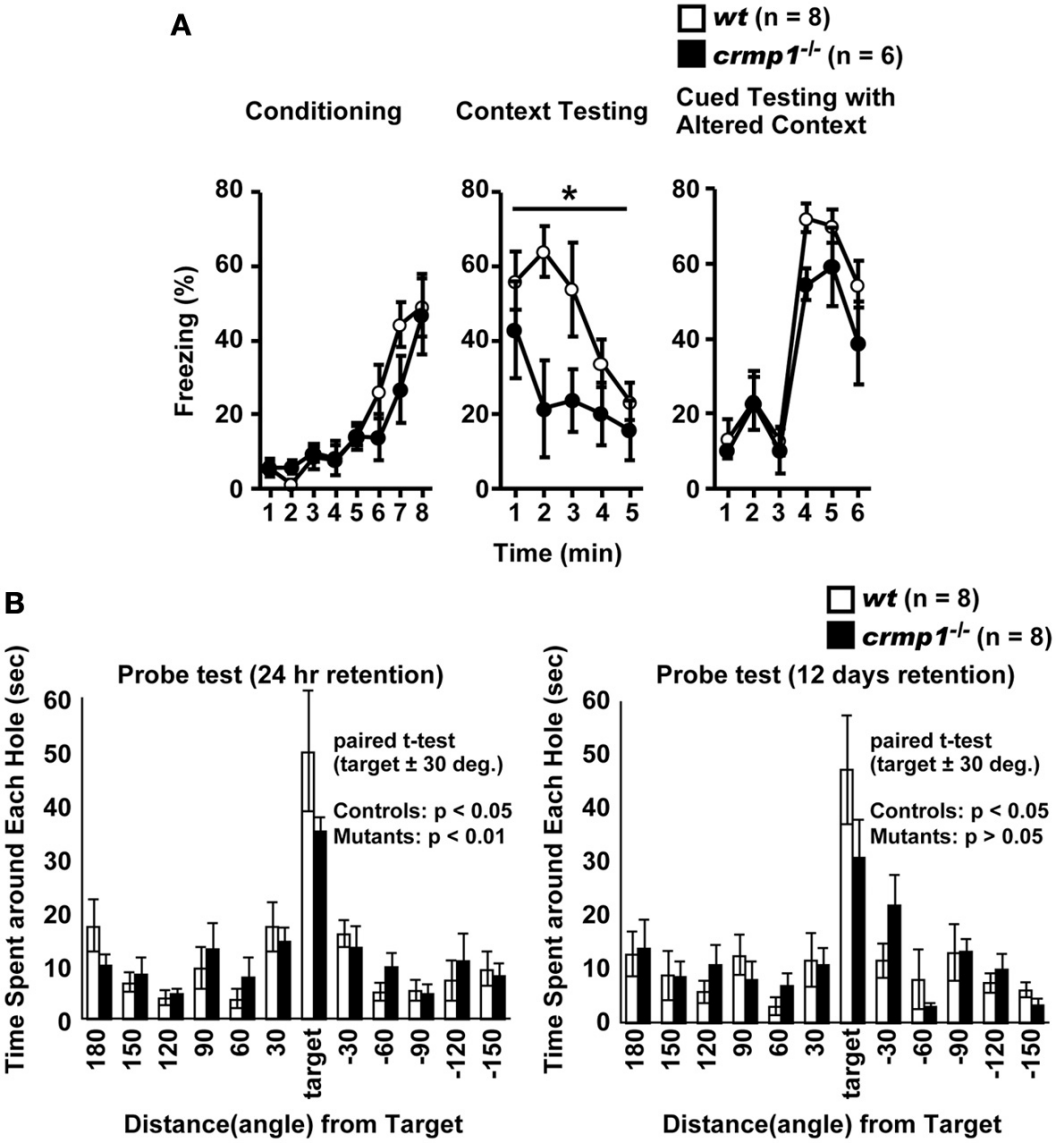
**Impaired learning and memory of *crmp1*^−/−^ mice**. **(A)** Contextual and cued fear conditioning test. Freezing rate during the training phase, contextual test, and cued test were calculated. The *p* values indicate genotype effect in two-way repeated measures ANOVA. **(B)** The probe trial conducted 24 h or 12 days after the last training in the Barnes maze test. Time spent around each hole was recorded. Time spent around target hole and holes adjacent to the target was compared by paired *t*-test. Data are shown as mean ± s.e.m. for the indicated numbers of mice. *p < 0.05.

To assess whether the loss of CRMP1 was associated with cognitive abnormalities, we performed various memory and learning tests with *crmp1*^−/−^ mice. In a contextual and cued fear conditioning test, *crmp1*^−/−^ mice did not show a significant difference in the levels of freezing during conditioning [Figure 3A; *F*_(1, 12)_ = 0.498, *p* = 0.4937]. In contrast, *crmp1*^−/−^ mice showed decreased levels of freezing in a contextual test but not in a cued test 1 day after conditioning [Figure 3A; contextual test, *F*_(1, 12)_ = 4.936, *p* = 0.0463; cued test, *F*_(1, 12)_ = 3.826, *p* = 0.0742]. Freezing during the context test is attributed to hippocampal or temporal lobe processes (Anagnostaras et al., 2001). Deficits in freezing during both the context and cued tests are indicative of amygdala dysfunction (Phillips and Ledoux, 1992; Amorapanth et al., 2000). Our finding suggests that the function of hippocampal and/or temporal lobe was selectively impaired in *crmp1*^−/−^ mice.

To examine the possible effect of CRMP1 deficiency on long-term spatial memory, we subjected mice to the Barnes maze test. Both *crmp1*^−/−^ and *wt* mice learned to locate the escape hole during the course of the training period (data not shown). Through the training trials, there were no statistical differences between *crmp1*^−/−^ and *wt* mice in latencies [*F*_(1, 14)_ = 0.47, *p* = 0.5044 by two-way repeated measures ANOVA] and errors [*F*_(1, 14)_ = 0.107, *p* = 0.7484 by two-way repeated measures ANOVA] to escape through the target hole. The probe trial was conducted 24 h after the last training session. Both *crmp1*^−/−^ and *wt* mice selectively located the correct target hole where the escape box had been, and both *crmp1*^−/−^ and *wt* mice spent significantly more time around the target hole compared to the holes adjacent to the target [Figure 3B; *wt*: *t*_(7)_ = 3.216, *p* = 0.0147; *crmp1*^−/−^: *t*_(7)_ = 6.891, *p* = 0.0002]. To assess the long-term retention of spatial memory in *crmp1*^−/−^ mice, we also conducted probe tests 12 days after the last training trial. Again, *wt* mice selectively located the correct target hole and spent significantly more time compared to the adjacent holes, but *crmp1*^−/−^ mice did not [Figure 3B; *wt*: *t*_(7)_ = 3.369, *p* = 0.0119; *crmp1*^−/−^: *t*_(7)_ = 2.325, *p* = 0.053]. These results indicate that *crmp1*^−/−^ mice were impaired in memory retention rather than memory recall. We also performed a T-maze forced alternation task, a task of working memory. The *crmp1*^−/−^ and *wt* mice did not show a significant difference (Table 1), suggesting normal working memory in *crmp1*^−/−^ mice.

### Impaired prepulse inhibition and effect of chlorpromazine in *crmp1*^−/−^ mice

**Figure 4:**
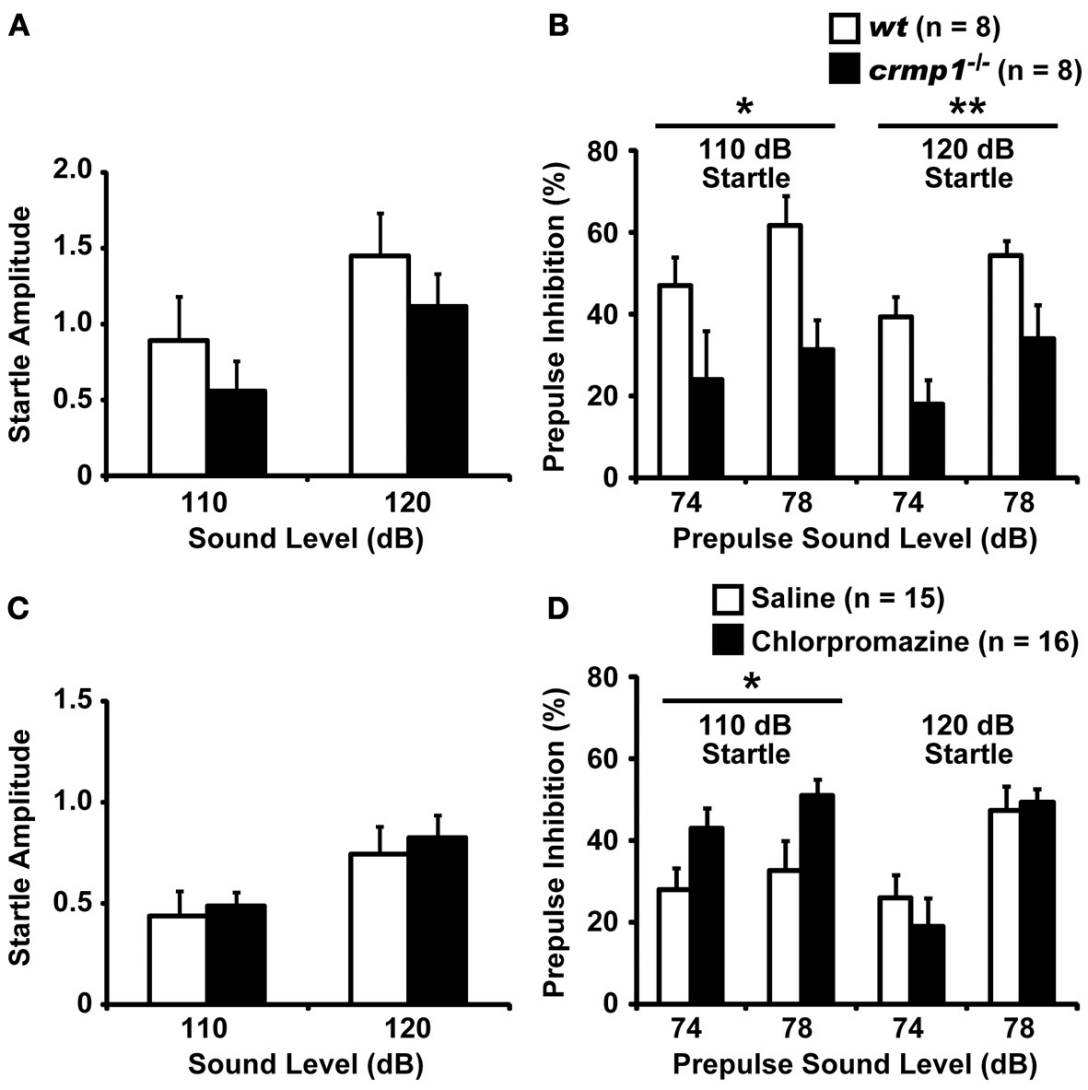
**Impaired prepulse inhibition and effect of chlorpromazine in *crmp1*^−/−^ mice**. **(A,B)** Comparison between *crmp1*^−/−^ and *wt* mice in prepulse inhibition test with the amplitudes of the startle response **(A)** and the percentage of prepulse inhibition **(B)**. **(C,D)** Comparison between chlorpromazine- and saline-treated *crmp1*^−/−^ mice in prepulse inhibition test with the amplitudes of the startle response **(C)** and the percentage of prepulse inhibition **(D)**. The *p* values indicate genotype **(A,B)** or drug **(C,D)** effect in two-way repeated measures ANOVA. Data are shown as mean ± s.e.m. for the indicated numbers of mice. ^*^p < 0.05, ^**^p < 0.01.

We compared the efficiency of sensorimotor gating in *crmp1*^−/−^ and *wt* mice as assayed by the prepulse inhibition test. The amplitudes of the startle response were similar in *crmp1*^−/−^ and *wt* mice [Figure 4A; *F*_(1, 14)_ = 0.937, *p* = 0.3495]. The percentage of prepulse inhibition was significantly decreased in *crmp1*^−/−^ mice [Figure 4B, 110dB, *F*_(1, 14)_ = 8.377, *p* = 0.0118; 120dB, *F*_(1, 14)_ = 9.819, *p* = 0.0073], suggesting impaired sensorimotor gating. Because disruption of prepulse inhibition is a behavioral feature used to model one aspect of schizophrenia that is shared by several other disorders (Braff and Geyer, 1990; Paylor and Crawley, 1997), we treated *crmp1*^−/−^ mice with chlorpromazine, a typical antipsychotic drug. Intraperitoneal injection of 1 mg/kg of chlorpromazine, which did not affect startle response [Figure 4C; *F*_(1, 29)_ = 0.1839, *p* = 0.6712], significantly rescued impaired prepulse inhibition with a 110-dB startle stimulus [Figure 4D; 110dB, *F*_(1, 29)_ = 5.361, *p* = 0.0279; 120dB, *F*_(1, 29)_ = 0.1559, *p* = 0.6959].

### Increased methamphetamine-induced release of dopamine in *crmp1*^−/−^ mice

**Figure 5:**
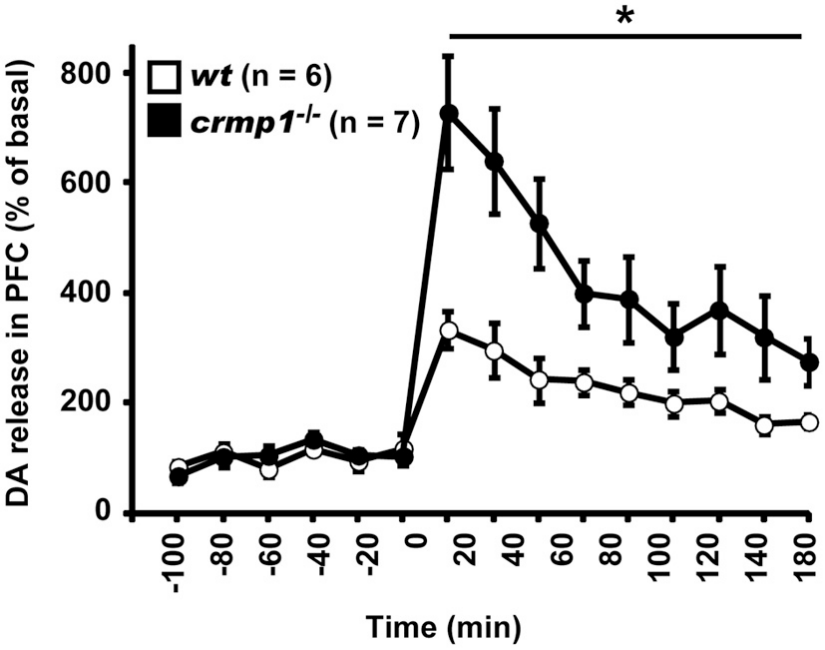
**Enhanced methamphetamine-induced release of dopamine (DA) of *crmp1*^−/−^ mice**. The extracellular concentration of DA in the prefrontal cortex of freely moving mice was determined by *in vivo* microdialysis. After collection of basal fractions, methamphetamine (1 mg/kg, i.p) was administrated at time 0. Dopamine concentration was expressed as a percentage of the average of that in the six baseline fractions before drug administration. Statistic analysis was performed before and after the drug administration. The *p* values indicate genotype effect in two-way repeated measures ANOVA. Data are shown as mean ± s.e.m. for the indicated numbers of mice. ^*^p < 0.01.

Because chlorpromazine is an effective antagonist of D2 dopamine receptors, it is possible that *crmp1*^−/−^ mice exhibit impaired dopaminergic function. To test this hypothesis, we performed *in vivo* microdialysis to examine the extracellular level of dopamine. Samples were collected from the prefrontal cortex of freely moving mice, and the extracellular concentration of dopamine was determined by high-performance liquid chromatography. We also measured the dopamine level after the injection of methamphetamine (1 mg/kg, i.p.). The mean basal levels of dopamine before drug administration were similar between *wt* and *crmp1*^−/−^ mice [7.57 ± 1.35 pg/ml in *wt* (*n* = 6), 8.71 ± 3.11 pg/ml in *crmp1*^−/−^ (*n* = 7), *F*_(1, 11)_ = 0.0955, *p* = 0.7584]. In contrast, the methamphetamine-induced increase in dopamine concentration was significantly greater in *crmp1*^−/−^ mice [Figure 5; *F*_(1, 11)_ = 9.7936, *p* = 0.0096]. These data suggest that CRMP1 deficiency resulted in an increase in methamphetamine-induced dopaminergic neurotransmission in the prefrontal cortex.

## Discussion

Schizophrenia can be understood, in part, as abnormalities of neuronal development (Bray et al., 2010). In this study, we demonstrated that knockout of *crmp1*, a key gene in neuronal development, caused aberrant behaviors such as increased locomotor activity, impaired emotional behavioral responses, and prepulse inhibition. Hyperactivity and impaired emotional behavior are key endophenotypes of many human psychiatric disorders, including schizophrenia (Gainetdinov et al., 2001), attention deficit hyperactivity disorder, and manic disorders (Paule et al., 2000), and it is also characteristic of rodent models of schizophrenia (Gainetdinov et al., 2001). The acute administration of methamphetamine in humans induces delusions and hallucinations reminiscent of those associated with schizophrenia. Furthermore, sensitivity to this drug is increased in individuals with schizophrenia (Lieberman et al., 1987) as well as in several mouse models that manifest schizophrenia-like behaviors (Paterlini et al., 2005; Pillai-Nair et al., 2005; Sakae et al., 2008). Furthermore, the *crmp1*^−/−^ mice exhibited an increase in methamphetamine-induced dopaminergic neurotransmission in the prefrontal cortex (Figure 5), whose dysfunction has been shown to be related to many neuropsychiatric disorders (Bray et al., 2010). The impaired prepulse inhibition, another typical endophenotype of schizophrenia (Braff and Geyer, 1990; Paylor and Crawley, 1997), of *crmp1*^−/−^ mice was rescued by the administration of chlorpromazine (Figure 4). These data suggest that the behavioral abnormality of *crmp1*^−/−^ mice is closely related to impaired dopaminergic signaling, which is one of the most common causes of schizophrenia (Ross et al., 2006). It is known that negative symptoms are not modeled equivalently to positive symptoms. In our behavioral test battery, the *crmp1*^−/−^ mice did not show an anhedonic or depressive-like behavior. Furthermore, these mice showed no abnormality in social interactions (Table 1). Thus, the *crmp1*^−/−^ mice exhibited some behavioral phenotypes that are related to the positive but not negative symptoms of schizophrenia.

Most rodent models of schizophrenia tend to replicate aspects of the positive symptoms of schizophrenia, such as hyperactivity, probably reflecting enhanced mesolimbic dopamine function (Breier et al., 1997; Laruelle et al., 1999). In rodents, chronic amphetamine administration induces a persistent sensitization, exaggerating the hyperactivity caused by acute amphetamine challenge (Featherstone et al., 2007), and there have been some reports of genetic models that show hyperdopaminergic functions (Paterlini et al., 2005; Pillai-Nair et al., 2005; Sakae et al., 2008). In addition to a hyperdopaminergic phenotype, *crmp1*^−/−^ mice show a poor dendritic trees and a low density of dendritic spines in the cerebral cortex (Yamashita et al., 2007). In human Schizophrenic patients, a marked reduction in dendritic spine density and glutamate receptor immunoreactivity is observed in cortical areas of schizophrenic subjects (Glantz and Lewis, 2000; Kolluri et al., 2005; Garey et al., 2006; Garey, 2010). Thus, the phenotypes of *crmp1*^−/−^ mice are consistent with those of schizophrenic patients either from a functional or morphological point of view, suggesting that *crmp1*^−/−^ mice may be a comprehensive model that more adequately replicates deficits in schizophrenic symptoms.

The underlying mechanism of the *crmp1*^−/−^ mouse phenotype is unknown. CRMP1 is phosphorylated by Cdk5, a major protein kinase that regulates dendritic spine maturation and dopamine signaling in neurons (Bibb et al., 1999; Morabito et al., 2004; Cole et al., 2006; Yamashita et al., 2007). Cultured cortical neurons from both *crmp1*^−/−^ and *cdk5*^−/−^ mice show abnormal dendritic spine morphology. In addition, the abnormal dendritic spine morphology in *crmp1*^−/−^ cultured cortical neurons is rescued by introduction of wt CRMP1 but not a CRMP1 mutant that cannot be phosphorylated by Cdk5 (Yamashita et al., 2007), demonstrating that CRMP1 is a major substrate of Cdk5 to regulate neuronal development. Thus the schizophrenia-related behaviors seen in *crmp1*^−/−^ mice may be caused by the disruption of Cdk5-CRMP1 signaling. In fact, mice treated with Cdk5 inhibitors and CA1-specific *cdk5* conditional knockout mice show decreased levels of freezing in a contextual test but not in a cued test (Fischer et al., 2002; Guan et al., 2011). This phenotype is similar to that seen in *crmp1*^−/−^ mice (Figure 3A). Interestingly, the expression level of p35, a neuron-specific activator of Cdk5, is decreased in both prefrontal cortex and hippocampus of schizophrenia post-mortem brains (Engmann et al., 2011).

The tyrosine phosphorylation of CRMP1 may also be involved in behavioral abnormalities of *crmp1*^−/−^ mice. CRMP1 is tyrosine phosphorylated by Fyn downstream of Reelin signaling (Yamashita et al., 2006). Reelin signaling also has been implicated in various neurodevelopmental disorders (Fatemi, 2005). The mRNA and protein expression of Reelin are decreased in patients with schizophrenia and bipolar disorder (Impagnatiello et al., 1998; Guidotti et al., 2000). Heterozygous *reeler* mutation mice, which show a 50% reduction in Reelin protein and mRNA, exhibit decreased prepulse inhibition (Tueting et al., 1999), as we observed in *crmp1*^−/−^ mice (Figure 4). Thus, it is possible that behavioral abnormalities of *crmp1*^−/−^ mice are related to CRMP1 phosphorylation at tyrosine residue(s).

Recently, several *crmp* genes (*crmp2*: 8p22-p21; *crmp3*: 10q26; *crmp4*: 5q32) were located to chromosomal regions associated with schizophrenia (Ross et al., 2006). Similar to that linkage study, *crmp1* or *crmp2* gene polymorphisms and abnormal CRMP1 or CRMP2 protein levels have been found in schizophrenia (Edgar et al., 2000; Johnston-Wilson et al., 2000; Hong et al., 2005; Beasley et al., 2006; Bader et al., 2012). In addition, aberrant accumulation of insoluble forms of CRMP1 is also observed in brains from schizophrenic patients (Bader et al., 2012). These findings may be inconsistent with our present finding that *crmp1*^−/−^ mice showed schizophrenia-related behaviors. The reasons for the apparent discrepancy between human and animal subjects are unknown. However, it is possible that the presence of these insoluble forms of CRMP1 may lead to the loss of function of CRMP1 in the human brains. In this case, our observation of behavioral abnormalities in *crmp1*^−/−^ mice may be consistent with findings obtained in specimens from schizophrenia patients (Bader et al., 2012). Interestingly, lymphoblastoid cell lines derived from schizophrenia patients show an abnormal level of CRMP1 expression, suggesting its potential role as a blood-based diagnostic marker (Bader et al., 2012). Taken together, these findings raise the possibility that CRMP1 may be involved in the pathophysiology in schizophrenia.

In conclusion, our results suggest that CRMP1 is associated with some of the symptoms of schizophrenia and/or some related disorders. Although future studies are need to determine whether *crmp1*^−/−^ mouse is a clinically relevant model of schizophrenia, our results suggest that *crmp1*^−/−^ mouse is useful in exploring basic mechanisms that may contribute to some of the symptoms of this neuropsychiatric disorder.

## Author contributions

Tsuyoshi Miyakawa and Yoshio Goshima designed the study and supervised the project; Naoya Yamashita, Aoi Takahashi, and Toshifumi Yamamoto carried out the experiments; Pappachan Kolattukudy provided *crmp1*^−/−^ mice; Keizo Takao supervised the behavioral experiments; and Naoya Yamashita and Yoshio Goshima wrote the manuscript.

### Conflict of interest statement

The authors declare that the research was conducted in the absence of any commercial or financial relationships that could be construed as a potential conflict of interest.
